# Supplementary material for: Rapid turnover of life-cycle-related genes in the brown algae
Source: Genome Biol. 2019 Feb 14;20:35. doi: 10.1186/s13059-019-1630-6 (PMC6374913; doi:10.1186/s13059-019-1630-6)
Supplement: Supplementary file 3 — Protein alignments, corresponding Gblocks html file and CDS sequences for the lineage conserved gametophyte-biased genes. (ZIP 2109 kb) [file 13059_2019_1630_MOESM3_ESM.zip › Con_GA_alignments/Ec-14_000390.aa_ali.fasta-gb.htm]

Ec-14\_000390.aa.fasta.trans.aa\_ali.fasta


## Gblocks 0.91b Results

Processed file: **Ec-14\_000390.aa.fasta.trans.aa\_ali.fasta**  
Number of sequences: **2**  
Alignment assumed to be: **Protein**  
New number of positions: **577** (selected positions are underlined in blue)

```
                         10        20        30        40        50        60
                 =========+=========+=========+=========+=========+=========+
Ec-14_000390     MEIG--TTNCDGGEQEWLRPAVPASRPTAPTESKRTSVLKHRTFAPKSFGRNKAPSGEGG
MSTRG.41755.1    MSKNGDRQHRDENEREWLRPAAAAP----SFESKRKSTLKRRTLAP-SFGRKAALSQEEE
                           ##############       ############### ###########  


                         70        80        90       100       110       120
                 =========+=========+=========+=========+=========+=========+
Ec-14_000390     KDDIEIAK-DDENFRNGLKRNIIHLSNLTRVNQEQLARLREQLKAKDHEIASLKALKENV
MSTRG.41755.1    TEANDFFRGDEEKFRNGLKRNIALLSNMTRVNQAEMARLREQLKAKDAEIMSLKAKFEEA
                          #################################################  


                        130       140       150       160       170       180
                 =========+=========+=========+=========+=========+=========+
Ec-14_000390     RQEDRQQDPPEKGPGPSERTNAGRCGPRKLVTYGKMVVTAGRLASEPRQRPEKAVAQPSE
MSTRG.41755.1    DLQET---VRDRDPGLSSSQNSARPRKEKLTTYANMAVMASKLTSHTRNRLTKAASEPSE
                              ###############################################


                        190       200       210       220       230       240
                 =========+=========+=========+=========+=========+=========+
Ec-14_000390     KETLETERAVAAMGAKHTEQQLLRASRDVRGQLERALQEAEALRADNIAFKTRVNVAEKE
MSTRG.41755.1    QDRVAEKMAVAAVGARHTEQQLLRANRDVRGQLDRALKEAAALRADNVGSKNRADLREKE
                 ############################################################


                        250       260       270       280       290       300
                 =========+=========+=========+=========+=========+=========+
Ec-14_000390     KAHLETRVALLSDEVKLQIAPLKALQLELTQTRSDTETMRQRLKEERRESRLRVSDSDKA
MSTRG.41755.1    KAILQARVAWLSDEVKLQTAPLLALQLELRQAKSDSEVMRQRLQEERKENRIRVGEMEKA
                 ############################################################


                        310       320       330       340       350       360
                 =========+=========+=========+=========+=========+=========+
Ec-14_000390     KSEAQQRIDLLEFDLNETAKACQRESLKASTFEGMYQEASTMATLLQAEVEQCRTDAKMI
MSTRG.41755.1    SVEAQQRGERLEFDLNEVAKACQRETSRAAKLDEMFQEASAAASSLREEVQQRKVDAKRM
                 ############################################################


                        370       380       390       400       410       420
                 =========+=========+=========+=========+=========+=========+
Ec-14_000390     AQELTRMQIESEKSSALVGLTPSLALSSPVDVGRAVGGVDGKEVTTGKLGEEQEAEIASL
MSTRG.41755.1    AQELSKIMLDSEKPNHLADSTPPAYFPLRGDGGEAVRAGEDQEGTKKQL-EEQRAEIATL
                 ################################################# ##########


                        430       440       450       460       470       480
                 =========+=========+=========+=========+=========+=========+
Ec-14_000390     KKIIAQLASEKSDSG---DAMKNLLEAHRREGEERERAFKEMQKKADSFAEENIGLRATI
MSTRG.41755.1    KTALAHVRLEHAKSDDTVKGMRQMQQMHRAEIAQGKRASSEAQDRAAFFSNENDRLRLAM
                 ##############      ########################################


                        490       500       510       520       530       540
                 =========+=========+=========+=========+=========+=========+
Ec-14_000390     EKQEEESERQRVEEDERHDRDIKREIILSQQNLDSASLRDRLCRTVDELCGAEEAGELVL
MSTRG.41755.1    EKQDEEQRKQ--QDEQRKERDAQVDKMASAQRVHHKSWRDRIRRTLEELCNAEEAGELVL
                 ##########      ############################################


                        550       560       570       580       590       600
                 =========+=========+=========+=========+=========+=========+
Ec-14_000390     TCQRCAKLFKDPHTMAPCGHTLCAGCCAGGDG----GMSAEDGTGRVPGGSGESPGSVTH
MSTRG.41755.1    TCLKCARLFKDPHIMAPCGHTLCADCCVGGDGGGDGGLPDEESSGWVLGGGGGE--AAKG
                 ################################    ################        


                        610       620       630       640       650       660
                 =========+=========+=========+=========+=========+=========+
Ec-14_000390     AEPECYLCGQQGRGATDRVACVGMAPNRALASVVMKFAFRRQLVQTLKEIGAALSGQGSV
MSTRG.41755.1    LPPTCKVCGERESQGVDKVGCVGMAPNRALAALVVKFAFRRKLLKTLKDLV---------
                   ##############################################            


                 
                 ========
Ec-14_000390     ILDSVDGV
MSTRG.41755.1    --------
```

```
Parameters used
Minimum Number Of Sequences For A Conserved Position: 2
Minimum Number Of Sequences For A Flanking Position: 2
Maximum Number Of Contiguous Nonconserved Positions: 8
Minimum Length Of A Block: 10
Allowed Gap Positions: None
Use Similarity Matrices: Yes
```

```
Flank positions of the 10 selected block(s)
Flanks: [11  24]  [32  46]  [48  58]  [70  118]  [134  409]  [411  434]  [441  490]  [497  572]  [577  592]  [603  648]  

New number of positions in Ec-14_000390.aa.fasta.trans.aa_ali.fasta-gb:  577  (86% of the original 668 positions)
```
